# Exploiting Plasma Exposed, Natural Surface Nanostructures in Ramie Fibers for Polymer Composite Applications

**DOI:** 10.3390/ma12101631

**Published:** 2019-05-18

**Authors:** Sameer F. Hamad, Nicola Stehling, Simon A. Hayes, Joel P. Foreman, C. Rodenburg

**Affiliations:** 1 Department of Materials Science and Engineering, The University of Sheffield, Sheffield S1 3JD, UK; nastehling1@sheffield.ac.uk (N.S.); j.foreman@sheffield.ac.uk (J.P.F.); c.rodenburg@sheffield.ac.uk (C.R.); 2 College of Engineering, University of Misan, Maysan 62001, Iraq; 3 Department of Multidisciplinary Engineering Education, The University of Sheffield, Sheffield S3 7RD, UK; s.a.hayes@sheffield.ac.uk

**Keywords:** ramie fibers, plasma treatment, nanoscale structures, microfibrils, surface wettability, LV-SEM

## Abstract

Nanoscale surface morphology of plant fibers has important implications for the interfacial bonding in fiber-polymer composites. In this study, we investigated and quantified the effect of plasma-surface modification on ramie plant fibers as a potential tool for simple and efficient surface modification. The extensive investigation of the effects of plasma treatment of the fiber surface nano-morphology and its effect on the fiber-polymer interface was performed by Low-Voltages Scanning Electron Microscopy (LV-SEM), infrared spectroscopy (FT-IR) analysis, fiber-resin angle measurements and mechanical (tensile) testing. The LV-SEM imaging of uncoated plasma treated fibers reveals nanostructures such as microfibrils and elementary fibrils and their importance for fiber mechanical properties, fiber wettability, and fiber-polymer matrix interlocking which all peak at short plasma treatment times. Thus, such treatment can be an effective in modifying the fiber surface characteristics and fiber-polymer matrix interlocking favorably for composite applications.

## 1. Introduction

Natural plant fibers are increasingly being used as a reinforcement in polymer matrix composites especially in applications where lighter and stronger materials are required, for instance in automotive and aerospace applications [1,2]. The unique characteristics of natural plant fibers such as high specific strength and stiffness with low density have motivated many researchers to use them as a replacement for synthetic fibers in polymer composites. For example, the specific modulus of ramie fibers (29–85 GPa/g cm^−3^) can be higher than thus of E-glass fibers (29 GPa/g cm^−3^) [3,4] and they boast wide availability, eco-friendliness, non-toxic nature, and less abrasiveness to plastic processing equipment [5,6].

Among lignocellulosic fibers, ramie fiber is one of the most widely used fibers in the field of polymer composites due to its high crystallinity and commercial availability [7,8,9] as well as being the longest and one of the strongest fine textile fibers [10,11]. Ramie fibers are single cell structures with length and diameter varying from 60–250 mm and 11–80 μm respectively [11]. The cell wall consists of oriented semi-crystalline cellulose microfibrils, a few nanometers in diameter, embedded in an amorphous matrix of lignin and non-cellulosic compounds such as hemicellulose and pectins [12]. The high tensile strength microfibrils form the fundamental structural unit of the cell wall and provide mechanical strength to the fiber [13]. Although the semi-crystalline cellulose microfibrils represent the main structural components of the fiber cell wall, the amorphous matrix of hemicellulose, lignin, and pectin also highly influence the fiber surface properties [14]. Based on previous studies, the presence of these non-cellulosic compounds on the surface of ramie fibers have resulted in relatively poor surface adhesion properties in different polymer matrices such as epoxy resin [7], polylactic acid (PLA) [15], and polypropylene [16]. Therefore, the challenge is to remove the amorphous constituents from the fiber surface and expose the crystalline cellulose microfibrils, thus roughening the surface and increasing the surface area available for mechanical and chemical bonding to polymer matrices [17].

Plasma treatment presents an attractive method for the surface modification of various materials. It offers many advantages compared with chemical treatments such as simplicity, low energy consumption, short treatment times, low cost, and it does not require water or any potentially hazardous chemicals [18,19,20]. Depending on the experimental conditions, plasma-surface modification of natural plant fibers can induce different effects such as surface cleaning, surface etching, crosslinking, and functionalization [21,22,23,24,25]. Most of the previous studies have used plasma treatment for surface cleaning, increasing the surfaces microroughness, and to create hydrophilic/hydrophobic surfaces [26,27,28]. However, very few studies [29,30] have used plasma treatment as a method to produce nanoscale structures for plant fibers which can roughen the fiber surface without affecting or changing the bulk properties. Moreover, and according to our knowledge, the relationship between those nanoscale surface structures and fiber mechanical properties has not been studied for the case of single (elementary) fibers. 

Therefore, in the present study we use plasma-surface treatment with the goal of maximizing the exposure of crystalline cellulose microfibrils by removing the amorphous constituents such as hemicellulose and lignin from the fiber surface, without adversely affecting the fiber mechanical properties. To identify and image the crystalline cellulose microfibrils within elementary fiber we present a detailed investigation of the surface topography and single fiber mechanical testing of ramie fibers under different durations of plasma treatment. We use Low Voltage Scanning Electron Microscopy (LV-SEM) to observe the presence and orientation of nanoscale structures (cellulose microfibrils) on the fiber surface after plasma treatment without any sample coating and subsequently investigate the relationship between exposed nanostructures, surface wettability, fiber-phenolic matrix interlocking, and single fiber mechanical properties, to define what constitutes optimal plasma treatment conditions for ramie fiber – phenolic polymer composites. 

## 2. Experimental Work

### 2.1. Materials

**Figure 1:**
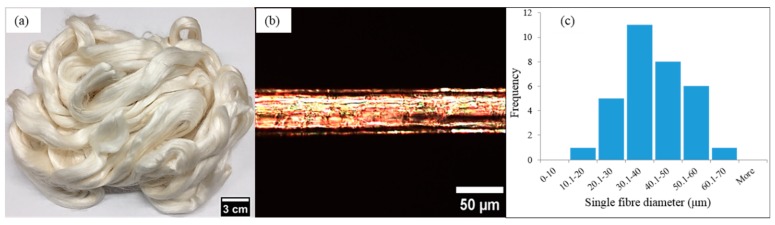
Ramie fibers (**a**) supplied bundle of ramie fibers, (**b**) optical microscopy image of single ramie fiber, and (**c**) histogram shows the most frequented diameter for single fibers, measured using optical microscopy images such as in (**b**).

Ramie fibers (Boehmeria nivea) were purchased from Wild Fibers store, Birmingham, UK and used as received for the experiments (Figure 1a). The most frequent single fiber diameter (Figure 1b) of the supplied fibers were in the range of 30–40 μm (Figure 1c). The fibers’ origin and extraction method is not disclosed by the supplier. For the polymer matrix, a resole commercial phenolic resin (Cellobond J2027X) and a slow acting acid catalyst (phencat 382) were supplied by Caleb Technical Products Ltd., Usk, UK, and used as a test liquid for the contact angle measurements.

### 2.2. Low Pressure Plasma-Surface Treatment

In this investigation, a Zepto plasma-surface cleaner (Diener electronic GmbH, Ebhausen, Germany) with a borosilicate glass cylindrical chamber (2.6 Liter) and power supply frequency of 40 kHz (Figure S1 in Supplementary Materials) was used to treat ramie fibers. The samples were prepared for the plasma treatment accordingly to the analysis to be performed: for the LV-SEM observations, single fibers were randomly selected and straightened to be held vertically on the SEM stub with a length of about 10 mm. The ends of each fiber were mounted on the stub with silver conductive paint and then inserted into the plasma chamber (Figure S2a in Supplementary information). For the Attenuated Total Reflectance-Fourier Transform Infrared spectroscopy (ATR-FTIR) characterization, a bundle of ramie fibers (30–50 mm long) were well distributed onto the sample holder (SEM stub), fixed on one side by carbon adhesive tape and then inserted into the plasma chamber (Figure S2b in Supplementary Materials). For the contact angle measurements, a single fiber with a length of about 40 mm was straightened and held vertically on a clean glass slide. A small piece of a card (0.4 mm thick) was placed in between the ends of each single fiber and the slide in order to prevent the fiber/slide contact and also to make sure the plasma uniformly treated all sides of the fiber (Figure S2c in Supplementary Materials). For the single fiber tensile testing, single fibers were randomly selected and mounted on a cardboard frame by using cyanoacrylate glue and then inserted into the plasma chamber (Figure S2d in Supplementary Materials). After that the plasma chamber was evacuated to 0.1 mbar of pressure to remove any contaminants from the chamber. Thereafter, the chamber was flooded with air and the gas flow rate was controlled by a flowmeter. Plasma was generated at 100 W and the chamber pressure was adjusted to 0.3 mbar. Ramie fibers were treated at four different durations: 1, 2, 3, and 4 minutes. All sample preparation and plasma treatments were performed in an environment of 22 ± 3 °C and 40% RH.

### 2.3. Surface and Cross-Section Observations of Ramie Fibers

A Nova Nano SEM 450 Scanning Electron Microscopy (FEI, Brno-Černovice, Czechia) was used to observe the fiber surface topography of untreated and plasma treated fibers. Moreover, single ramie fibers were cryo-fractured in the liquid nitrogen to observe the fiber cell wall structure by LV-SEM. The fracture surfaces of tensile test samples were also observed by LV-SEM. Natural plant fibers are not conductive materials; therefore, the observations were performed using a low accelerating voltage (1 kV) to avoid fiber charging. The images were collected using a through lens detector (TLD) at around 4.5 mm working distance with a beam deceleration of 2000 V. Fast Fourier Transform (FFT) image analysis method in ImageJ was used to observe the nanoscale structure orientation on the fiber surface.

### 2.4. Attenuated Total Reflectance-Fourier Transform Infrared Spectroscopy (ATR-FTIR)

A PerkinElmer FTIR spectrometer (PerkinElmer, Waltham, MA, USA) was used to investigate the surface chemical compositions of untreated and plasma treated ramie fibers in single reflection diamond ATR accessory with an angle of incidence of 16° from the perpendicular. Typically, the depth of light penetration in this technique is a few microns, depending on the wavelength, the angle of incidence, the refractive indices, and the sample [31]. In this study, the test was conducted at room temperature (22 ± 3 °C) with a wavenumber range between 4000 cm^−1^ to 600 cm^−1^ and the average of scan repetitions was 8 scans for each sample (untreated and plasma treated fibers) at 2 cm^−1^ of resolution.

### 2.5. Wettability Measurements

To evaluate the effect of plasma treatment on the fiber surface wettability, the single-fiber drop technique was used using drop-shape analyzer instrument (DSA 100-Kruss, Hamburg, Germany). Phenolic resin and its catalyst (phencat 382) were mixed in the ratio of 100:5 w/w and then used as a test liquid. This kind of resin is usually used as a matrix in polymer-fiber composites [32]. A 5 μL droplet was placed on the fiber surface using a microliter syringe. The drop shape was fitted with the Young-Laplace method and the angles were measured after 30 s of placing a drop onto the fiber surface. The angle values were taken as the average of at least 3 measurements obtained along the fiber surface. However, this is not a standard contact angle measurement as contact angle on single fibers is experimentally difficult, almost impossible to measure. This is due to the high curvature variation at the interface [33]. Therefore, to fully assess the surface wettability and surface adhesion properties in the context of composites, we have developed the experiment by curing the samples at 80 °C for 3 h using oven (Thermo Scientific Heraeus, Waltham, MA, USA). Thereafter, the fiber wetting area of the cured samples was observed using optical microscopy (Nikon Eclipse LV150, Tokyo, Japan). In addition, the fiber/matrix interface was also observed using LV-SEM for all differently cured samples.

### 2.6. Tensile Test of Single Ramie Fibers

A tensile testing machine (Zwick Roell, Ulm, Germany) with a 500 N load cell was used to test the mechanical tensile properties of single ramie fibers. Thirty isolated single fibers were prepared for untreated and plasma treated fibers at different times (1, 2, 3, and 4 min). Each single fiber with a gauge length of 5 mm was mounted on a cardboard frame by using cyanoacrylate glue. The fibers were carefully glued in the exact center of the cardboard. Thereafter, the sample was clamped onto the testing machine and just before the beginning of each test, the supporting side of the card was carefully cut off. The samples were tested at a constant crosshead displacement rate of 40 %/min and according to the ASTM D 3822-01 standard. The tests were carried out at room temperature (22 ± 3 °C). During the test, the force-strain values were recorded, and these values were used to measure the fiber tensile properties (ultimate strength and Young’s modulus). Only samples that broke in the middle of their gauge length were used to calculate the tensile strength and Young’s modulus, whereas the fibers that broke near to the glue clamp or card frame were not used in the calculations. 

**Figure 2:**
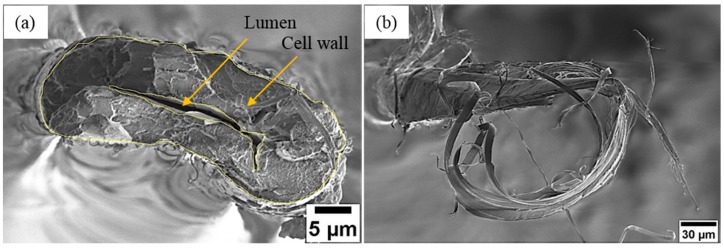
LV-SEM images of the fractured surface of single ramie fibers under tensile load (**a**) example of flat cross-section end and (**b**) example of single fiber split into fibrils. More SEM images are shown in the Supplementary Materials (Figures S3 and S4).

The cross-sectional area measurements highly affect the final tensile properties of single fibers [34]. Therefore, in this study, two different methods were used to determine the cross-sectional area:

1. Before testing the fiber, images at different locations along the fiber gauge length were taken using an optical microscope (Nikon Eclipse LV150, Tokyo, Japan). The cross-section of each single fiber was assumed to be circular. The fiber diameter was directly measured from the images and hence the cross-sectional area of each single fiber was calculated from the average of three apparent fiber diameter measurements (30 samples for each treatment time).
2. As the single fiber failure is elastic without any signs of plastic deformation, it can be assumed that the cross-sectional area of the fractured fibers has not changed significantly after the test. Therefore, after testing, LV-SEM was used to observe the cross-section area of the fractured fibers. Fractured fibers with a flat and clear cross-section end (Figure 2a) were selected for the cross-section area calculations whereas fractured fibers that split into fibrils (Figure 2b) were not included in the results as accurate area measurements were not possible for these samples. Thereafter, the collected LV-SEM images were used to calculate the actual cross-sectional area of the fractured fibers by using image J software. The hollow structure (lumen) can be clearly seen in Figure 2a, excluded from the total area. The sample size for each treatment time varied depending on fracture surface end (Table S1 in the Supplementary Materials).

## 3. Results and Discussion 

### 3.1. Microstructure of Single Ramie Fiber

**Figure 3:**
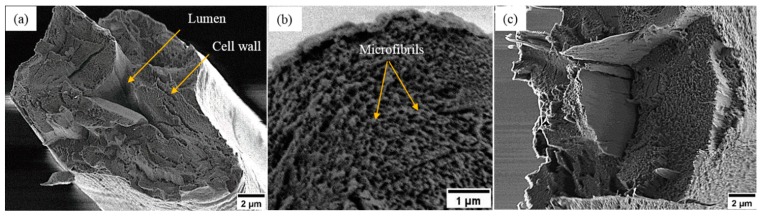
LV-SEM images of the cross-section of ramie fiber (**a**) overview of the cryo-fractured single ramie fiber, (**b**) the cryo-fractured cell wall, (**c**) the tensile fractured cell wall, more SEM images are shown in the Supplementary Materials (Figure S5).

The as received fibers were cryo-fractured to investigate the fiber cross-section structure by LV-SEM (Figure 3a). The LV-SEM observations highlight that the cross-section of single ramie fiber is irregular in shape with a central lumen surrounded by a thick cell wall. The cross-section view also shows that the cell wall structure of ramie fiber is composed of multiple layers. However, the structure of ramie fiber shown in Figure 3a is the typical structure of ramie fibers and most of natural plant fibers such as flax, hemp, kenaf and nettle fibers [35]. It can also be seen from the detailed investigation of the cell wall in Figure 3b that it mainly consists of bright nanoscale structures, which are expected to be crystalline cellulose microfibrils embedded in non-crystalline regions of hemicellulose and lignin. In addition, the cell wall structure of the tensile fractured sample (Figure 3c) shows the same features as in Figure 3b, indicating these bright nanoscale features are not cryogenic surface artefacts. These nanoscale features (Figure 3b) appear to be in the range of 10–40 nm in diameter and are thus consistent with crystalline cellulose microfibrils [36].

### 3.2. Surface Morphology Analysis

**Figure 4:**
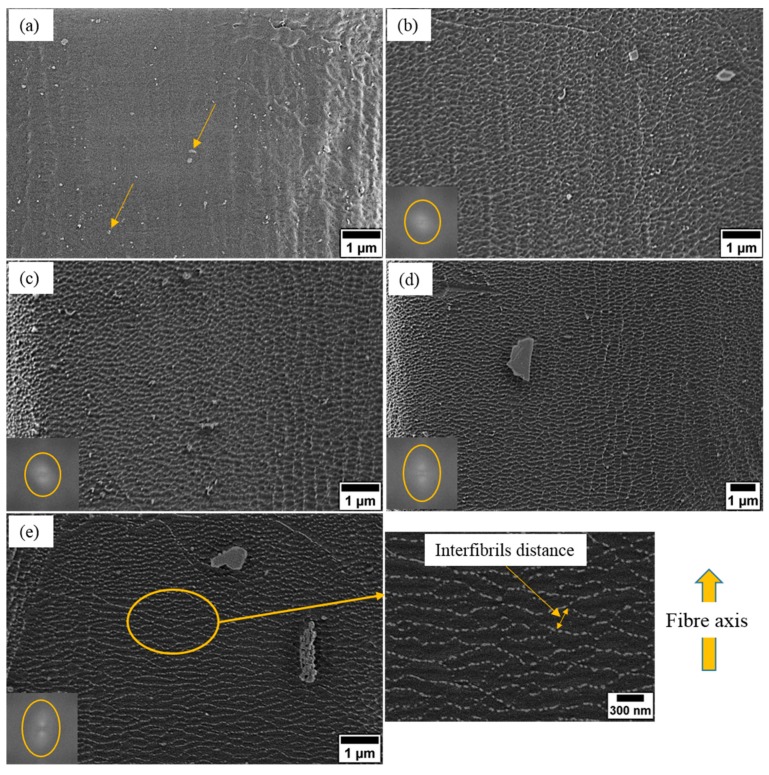
LV-SEM images of ramie fibers with their FFT images (bottom left) (**a**) untreated (yellow arrows point to impurities), and plasma treated for (**b**) 1, (**c**) 2, (**d**) 3, and (**e**) 4 min treatment duration with surface details marked by the yellow circle.

**Figure 5:**
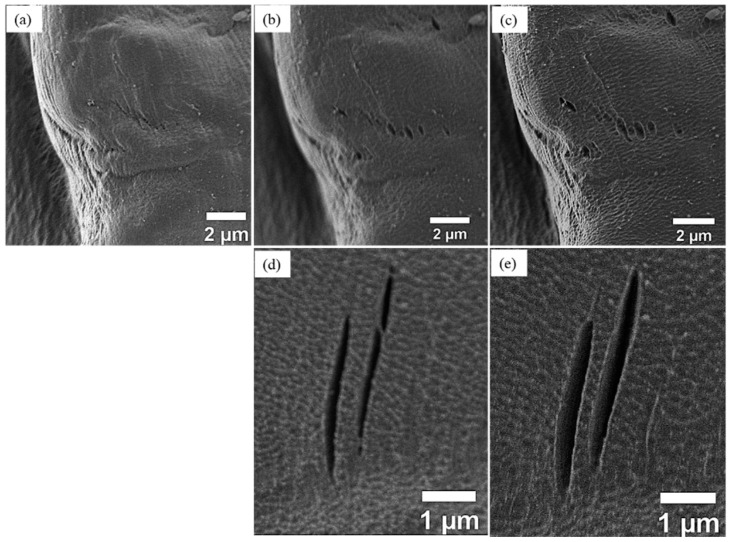
LV-SEM images of ramie fibers show the influence of plasma treatment on the pre-existing holes and cracks on the fiber surface, (**a**) untreated, (**b**,**d**) 2 min, (**c**,**e**) 3 min.

The surface morphology of untreated and plasma treated ramie fibers are shown in Figure 4. It can be visually verified that the surface roughness of the untreated fiber (Figure 4a) varies locally, with roughness at different scales. For instance it is rough on the microscale as shown in Figure 4a (right edge) and even more pronounced in Figure 5a whereas it is smooth and almost homogenous on the nanoscale (Figure 4a center) which is due to the primary amorphous layer that consists of waxes, pectins, and proteinaceous material [15,37,38]. However, 1 min of plasma treatment (Figure 4b) reveals nanoscale bright structures on the fiber surface, which lead to a rougher surface. These nanoscale structures are randomly oriented on the fiber surface as indicated by FFT image in Figure 4b (bottom left). It can also be seen from Figure 4b that most of the impurities have been removed from the fiber surface when compared to the untreated surface (arrows in Figure 4a). It has been reported that polymer chains in the amorphous state can be etched by plasma more easily than in the crystalline state [29,39]. Therefore, we attribute these nanoscale features to the crystalline cellulose microfibrils which remain after selective etching of the amorphous portions by plasma treatment. Moreover, these nanoscale structures have a similar diameter (10–40 nm) to those appear on the cross-section. Besides, it has been reported in the literature that the nanoscale surface features of plasma treated ramie fibers show secondary electron emission characteristics distinct from the matrix, further supporting the hypothesis that they are crystalline features in a more amorphous matrix [40]. 

When increasing the plasma treatment time to 2 min, those bright nanostructures (microfibrils) are seen more clearly on the fiber surface with their increased height indicating a large amount of amorphous material has been removed, leading to an increased surface roughness as presented in Figure 4c. Such effects contribute effectively to the improvements of the fiber surface wettability and the fiber mechanical properties (discussed in Section 3.4 and Section 3.5).

However, longer plasma treatment durations (3 and 4 min) leads to fewer, more isolated microfibrils as well as increases in the interfibrillar distance, especially after 4 min of plasma treatment as shown in Figure 4d,e. Besides, the FFT images (bottom left) in Figure 4d,e show that the microfibrils were aligned differently from those in Figure 4b,c. Therefore, we assume that most of the outer layer of the primary cell wall having been etched by plasma from the fiber surface, such that the inner face of the primary wall is exposed. According to our previous study [41] the thickness of the primary cell wall of ramie fiber is approximately 100 nm. Moreover, it has been reported that microfibrils are agglomerates of elementary fibrils with diameter of 3.5 nm [42]. Based on the sizes of nanofeatures (~ 4.5 nm) being very close into the reported diameter of elementary fibrils [42] in Figure 3e (surface details), we assume that after 4 min of plasma treatment the microfibrils have been broken up into their elementary fibrils. This can also explain the observed isolated appearance of fibrils and the increase in the interfibrillar distance after 4 min of plasma treatment. Such effects were found to have a major role in determining the fibers mechanical properties (see Section 3.5).

According to our LV-SEM observations, the plasma treatment is not creating holes or cracks on the fiber surface even after 4 min treatment duration. However, it is also important to mention that the LV-SEM observations in this work indicated that any pre-existing holes or cracks became wider and deeper as the treatment duration increases as shown in Figure 5. 

### 3.3. Surface Chemical Structure Analysis

**Figure 6:**
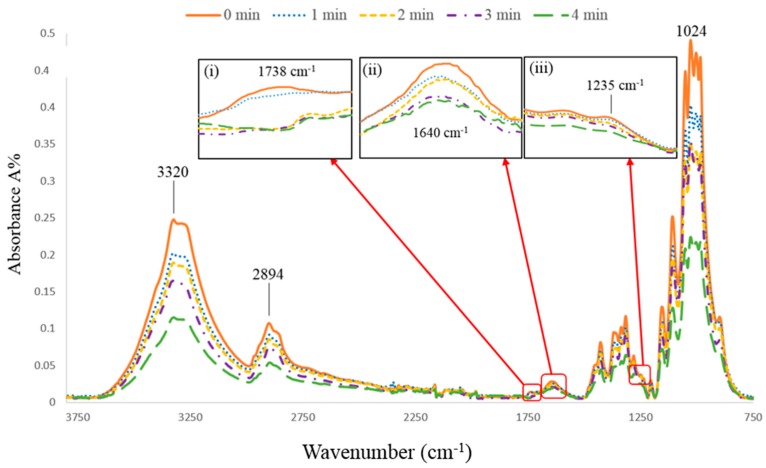
ATR-FTIR spectra of untreated and plasma treated ramie fibers at with enlarge sections of (i) carbonyl (C=O) stretching in cellulose and hemicellulose, (ii) water in crystalline cellulose, and (iii) the C–O vibration of esters, ethers, and phenolic groups.

The ATR-FTIR absorption spectra of untreated and plasma treated ramie fibers are shown in Figure 6. All spectra show many absorption bands that are mostly related to specific characteristic groups of the fiber components such as cellulose, hemicellulose, lignin, and waxes. Table 1 shows the standard [43,44,45,46] and the observed peak positions of ramie fibers, as well as the changes in the peak intensities after plasma treatment. However, very limited changes have been observed in the ATR-FTIR spectra of the plasma treated fibers, in comparison to the untreated fibers. The explanation for this is that the surface chemical changes after plasma treatment are expected to be limited to the topmost layer of the fiber surface while the depth of chemical information obtained by ATR-FTIR reported to be well below 1 μm the surface of the material being analyzed [47]. This further proves that short time of plasma treatment did not alter the fiber bulk properties.

The changes for the plasma treated fibers were observed in two main absorption cellulose bands (3220 cm^−1^ and 1024 cm^−1^), particularly after 4 min of plasma treatment. They are characteristic bands of the hydroxyl groups (O–H) present in the cellulose structures [44]. The reduction in the intensity of these two bands could be due to the partial removal of the cellulose microfibrils from the fiber surface after 4 min of plasma treatment, which is also in line with our LV-SEM observations. Also, changes were observed in the intensity of band at 1738 cm^−1^ after 2 min of plasma treatment (Figure 6(i)). This characteristic band corresponding to the carbonyl (C=O) stretching of acetyl groups in cellulose and hemicellulose [43,44]. The reduction in the intensity of this band after plasma treatment may be due to the removal of the amorphous cellulose and hemicellulose from the fiber surface [48]. In addition to the reduction of the intensity band of 1738 cm^−1^ after plasma treatment, it can also be seen there is a much narrower peak for the plasma treated fibers (2, 3, and 4 min) which might be due to the remaining of the crystalline cellulose [44]. Moreover, the peak near 1640 cm^−1^ is assigned to the absorbed water in crystalline cellulose [44]. This peak is found to decrease gradually with plasma treatment (Figure 6(ii)). Morshed et al. [44] and Sinha [45] suggested that the decreasing in the intensity of the band at 1650 cm^−1^ for the plasma treated fiber is probably due to the temperature effect and bond cleavage with plasma to form free radicals. The band near 1235 cm^−1^ is possibly due to the (C–O) vibration of esters, ethers, and phenolic groups related to the presence of waxes on the fiber surface and the decreasing of this band after plasma treatment (Figure 6(iii)) is probably due to the removal of waxes from fiber surface [46]. 

### 3.4. Surface Wettability of Fibers

**Figure 7:**
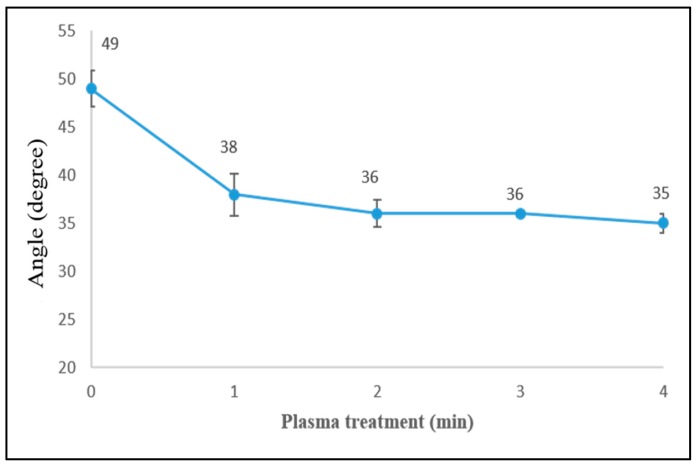
Angle values of single ramie fibers (plasma treated at various times) and phenolic resin.

The fiber surface changes that occur due to the plasma treatment lead to changes in the surface wetting characteristics, which were quantified by single fiber-liquid angle measurements. Generally, surfaces with smaller contact angle (less than 65°) are considered to be wettable surfaces and hydrophilic properties (high surface energy) [49], and a liquid drop tends to spread across the surface. Figure 7 shows the angle values between a single ramie fiber (untreated and plasma treated at various times) and phenolic resin. It can be seen that the angle of untreated fibers was 49° ± 1.9, while that of 1 min plasma treated fibers shows a smaller angle, 38° ± 2.2. However, no further clear decrease is observed with increasing treatment time (Figure 7). Modifications in the surface characteristics such as surface roughness and surface chemistry cause changes in the surface wettability. It has been reported that hydrophilic materials will appear more wettable as the surface roughness increases, while more hydrophobic in hydrophobic materials [50]. Therefore, the observed enhancement of the fiber surface wettability after plasma treatment can be attributed to increased surface roughness and greater exposure of crystalline cellulose microfibrils as well as the removal of the amorphous materials such as lignin and waxes from the fibers outermost layer (observed by LV-SEM and ATR-FTIR and discussed in Section 3.2 and Section 3.3). In addition, plasma treatment often introduces more polar groups such as hydroxyl groups on the fiber surface by oxidation of cellulose, which could also improve the fiber surface wettability.

**Figure 8:**
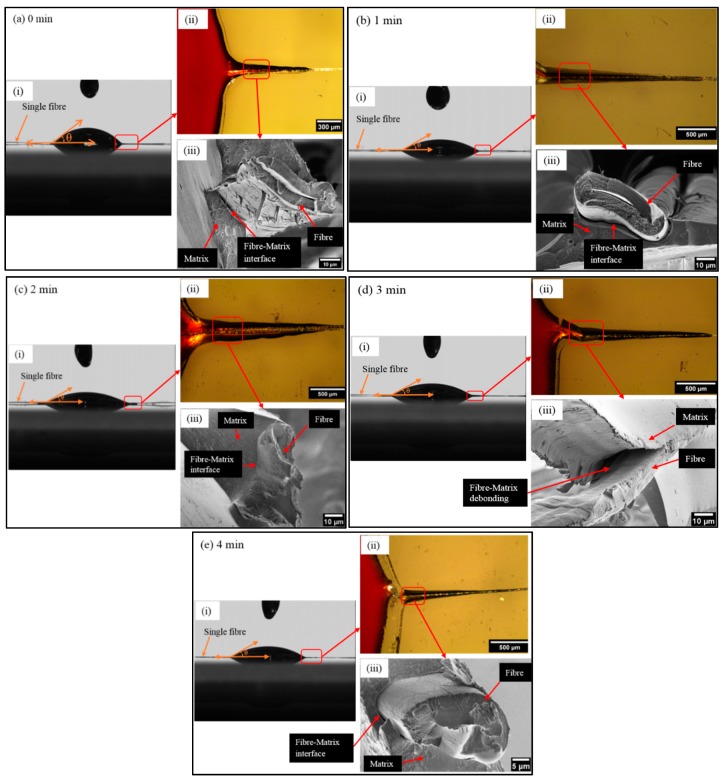
Phenolic resin drops on single ramie fibers plasma treated at various times: (**a**) 0 min, (**b**) 1 min, (**c**) 2 min, (**d**) 3 min, and (**e**) 4 min. Subfigures display: (i) measured angle of single ramie fiber with phenolic resin, (ii) optical microscopy image of single ramie fiber wetted by phenolic resin after curing, and (iii) LV-SEM image of single ramie fiber/phenolic resin interface.

The surface tension forces between fiber and phenolic resin enable the resin to spread across the fiber surface, as shown in Figure 8a(ii). These forces are highly dependent on surface roughness, crystallographic orientation, and chemical composition [50]. Therefore, for the plasma treated fibers (Figure 8b(ii)), there was a significant increase in the wetting area compared to the untreated fiber, which also correlates with the angle values. Moreover, the interfacial bond strength between fibers and matrix is an important factor in controlling the final mechanical properties for composites. Therefore, here we investigated the fiber-matrix interface using LV-SEM for all different samples. Figure 8a(iii) shows the untreated fiber-matrix interface. The defibrillation of the fiber surface and the long fiber pull out as well as a noticeable gab between fiber and matrix can be clearly seen, which indicates the poor surface adhesion between fiber and matrix. This could be due to the surface smoothness and the non-uniform fiber surface which results in fiber surface defibrillation and poor mechanical interlocking between fiber and matrix. However, the 1 min of plasma treated fiber (Figure 8b(iii)) shows shorter fiber pull out than the untreated fiber and more uniform fiber surface without defibrillation. This could be due to the improved surface roughness that is caused by plasma treatment, as well as the removal of the non-cellulosic compounds such as hemicellulose and waxes from the fiber surface. After 2 min of plasma treatment (Figure 8c(iii)), the interface between the phenolic matrix and the ramie fiber is almost continuous. The rough surface with greater exposure of crystalline cellulose microfibrils after 2 min of plasma treatment (observed by LV-SEM (Section 3.2)) enlarge the interfacial area, which may improve the fiber-matrix interaction. After 3 min of plasma treatment (Figure 8d(iii)), there was a fiber-matrix debonding and fiber pull out, which indicates the poor fiber-matrix interlocking. Besides, 4 min of plasma treatment also showed a fiber pull out and a gap between fiber and matrix (Figure 8e(iii)). These results were in a good agreement with the LV-SEM and ATR-FTIR analysis, which revealed that after a long time of plasma treatment (3 and 4 min) there was a partial removal of the cellulose microfibrils from the fiber surface resulting in fewer nanostructures and hence smaller interface area. 

### 3.5. Single Fiber Mechanical Properties

**Figure 9:**
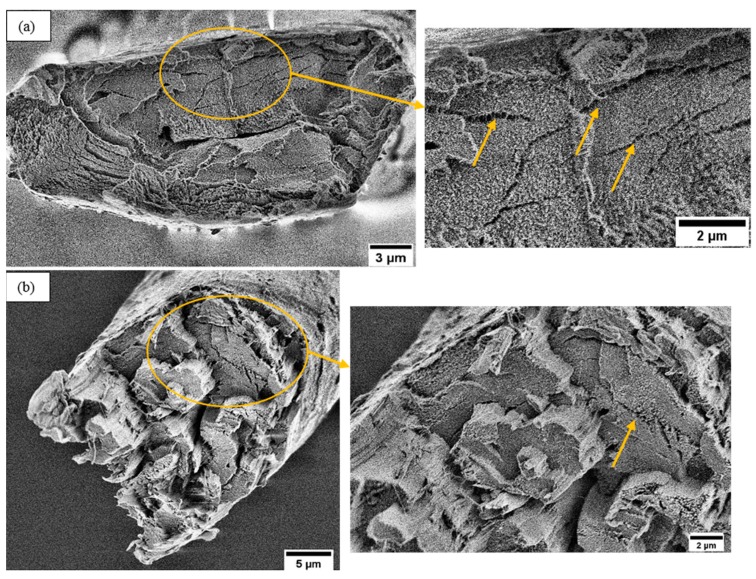
LV-SEM images of the fractured surface of single ramie fibers under tensile load (**a**) overview of a flat fractured surface with a detail cell wall structure inside yellow circle (**b**) overview of an irregular fractured surface with a detail cell wall structure inside yellow circle. Yellow arrows highlight cracks.

The fractured surface morphology of the tensile test of single ramie fibers is shown in Figure 9. It can be seen that some of the fractured fibers showed a very flat end surface (Figure 9a) whereas some other fractured fibers showed different fiber cells have fractured in different planes (Figure 9b). This behavior of fiber cells is probably due to the variability in the fiber cell strength and due to cell wall flaws [51]. It has been found that the variations in the fiber cell wall fracture can strongly influence the final fiber properties [41]. In addition, multiple cracks were observed (arrows in Figure 9) on the fractured surfaces, which is probably due to the stress during testing passing through weak regions which are composed of amorphous hemicellulose and lignin.

**Figure 10:**
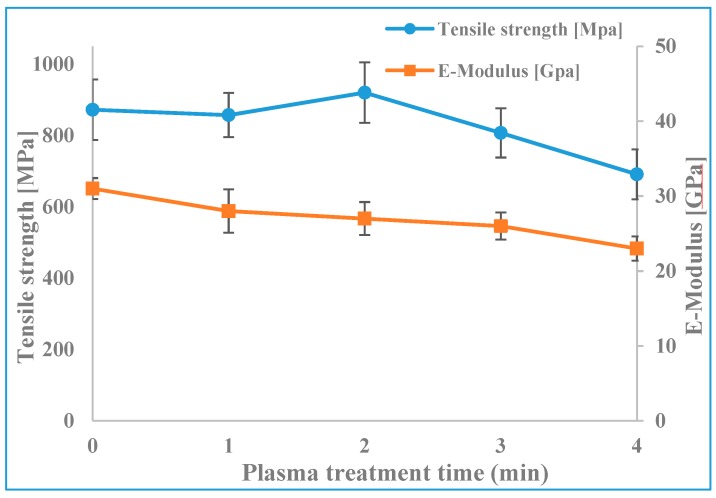
The tensile strength and Young’s modulus of untreated and plasma treated fibers, values based on actual cross-sectional area measurements from SEM images.

To determine to what extent plasma treatment durations affect the mechanical properties of ramie fibers, tensile properties (strength and Young’s modulus) were measured. Using the two different methods to measure the cross-sectional area of single fibers described in the experimental section, the strength and Young’s modulus with plasma treatment time were evaluated. The non-circular cross-section of single ramie fiber is clearly illustrated in Figure 9a, hence, assuming a circular cross-section in measuring the cross-sectional area many not be sufficient to accurately assessing the single fiber properties (Figure S6 in Supplementary Materials). Even though it means a reduction in sample size, we present the tensile results calculated using the second method using LV-SEM images to yield more accurate results. The results using fiber diameter measurements follow broadly the same trend, albeit with erroneous absolute values for the strength and Young’s modulus, and are shown in the Supplementary Materials. Based on the LV-SEM measurements, the average tensile strength and Young’s modulus of untreated fibers were 872 MPa and 31 GPa respectively (Figure 10). However, it can be seen that there are no significant changes in between the average values of tensile strength and Young’s modulus for 0, 1 and 2 min of plasma treatment which confirms that the short time of plasma treatment did not change the fiber bulk properties. With longer plasma treatment durations (3 and 4 min), the tensile strength and Young’s modulus values were found to decrease gradually. This could be due to the widening of the initial cracks and holes on the fiber surface, and also it might be due to the removal of most of the non-cellulosic binding materials as well as the partial removal of the cellulose microfibrils from the fiber surface, as indicated by LV-SEM results in Section 3.2. Moreover, the LV-SEM results presented above show that after 4 min of plasma treatment the microfibrils were broken up into their individual elementary fibrils with a clear increase in the interfibrillar distance. This could also provide an explanation for the low tensile properties after 4 min of plasma treatment.

## 4. Conclusions

Plasma-surface treatment of ramie fibers has created surface nanoscale structures after only 1 min of treatment. We identified these nanostructures to be crystalline cellulose microfibrils and report for the first time direct observation of the elementary fibrils. Exposing cellulose microfibrils resulted in a nanoscale surface roughness which increases the surface area and led to better wettability and fiber-matrix interaction. The use of plasma-surface modification could have immense benefits in the production of fiber-polymer composites with good interface integrity and could pose a big step forward in replacing fossil-fuel-based fibers.

## Figures and Tables

**Table 1:** Standard [43,44,45,46] and observed ATR-FTIR absorption peak position for ramie fibers.

Structural Bond	Associated Components	Standard Peak Position (cm^−1^)	Measured Peak Position (cm^−1^)	Effect of Plasma Treatment
OH stretching	Cellulose	3200 ~ 3600	~ 3320	Started to decrease after 3 min
CH2 stretching	Cellulose and hemicellulose	~ 2925	2894	None
C=O stretching	Cellulose and hemicellulose	1725 ~ 1750	1738	Decreased after 2 min
H–O–H bending	Absorbed water	~ 1650	1640	Decreased gradually with treatment time
CH2 deformation	Lignin	1435 ~ 1480	1423	None
CH3 bending	Lignin	1340 ~ 1390	1354	None
CH2 wagging	Lignin	~ 1320	1316	None
C–O stretching	Waxes	1275 ~ 1185	1235	Decreased gradually with treatment
C–O stretching	Cellulose	1160 ~ 1000	1094	None
O–H deformation	Cellulose	1080 ~ 1030	1024	Started to decrease after 3 min
β-Glucosidic linkage	Hemicellulose	~ 885	898	None

## Supplementary Materials

The following are available online at https://www.mdpi.com/1996-1944/12/10/1631/s1, Figure S1: Low pressure plasma - Diener electronic Zepto, Figure S2: Sample preparation (a) SEM sample, (b) FTIR sample, (c) contact angle sample, and (d) single fibre tensile testing sample, Figure S3: Examples of SEM images of the fractured surface of single ramie fibres show the flat and clear fracture end after tensile testing, Figure S4: Examples of SEM images of the fractured surface of single ramie fibres show the irregular fracture end after tensile testing, Figure S5: SEM images of the cell wall of ramie fibres after tensile testing show the microfibrils, Figure S6: The tensile strength and Young’s modulus of untreated and plasma treated fibres, values based on fibre diameter measurements (assuming circular cross section), Table S1: The sample size for each fibre (untreated and plasma treated) that used to determine the tensile strength and Young’s modulus using the actual cross sectional area.
