# Supplementary material for: Exploiting Plasma Exposed, Natural Surface Nanostructures in Ramie Fibers for Polymer Composite Applications
Source: Materials (Basel). 2019 May 18;12(10):1631. doi: 10.3390/ma12101631 (PMC6566196; doi:10.3390/ma12101631)
Supplement: Supplementary file 1 [file materials-12-01631-s001.pdf]

Supplementary Information: Exploiting Plasma Exposed, Natural Surface Nanostructures in Ramie Fibers for Polymer Composite Applications

Sameer F. Hamad[1,3*], Nicola Stehling[1], Simon A. Hayes[1], Joel P. Foreman[1], C. Rodenburg[1]

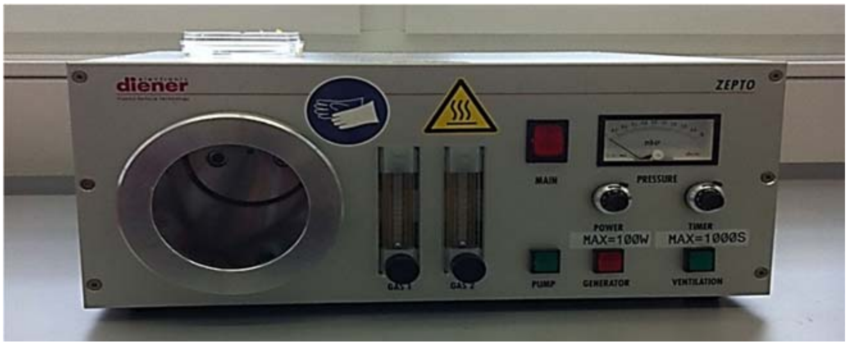

Figure S1. Low pressure plasma - Diener electronic Zepto.

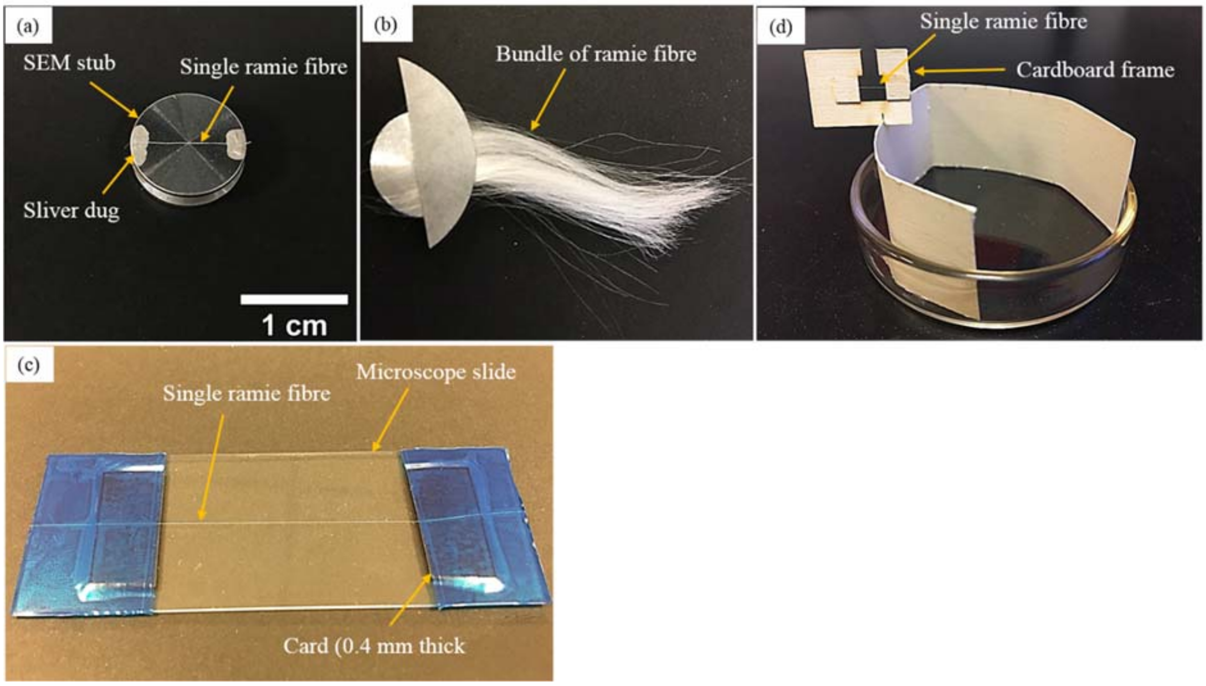

Figure S2. Sample preparation (a) SEM sample, (b) FTIR sample, (c) contact angle sample, and (d) single fibre tensile testing sample.

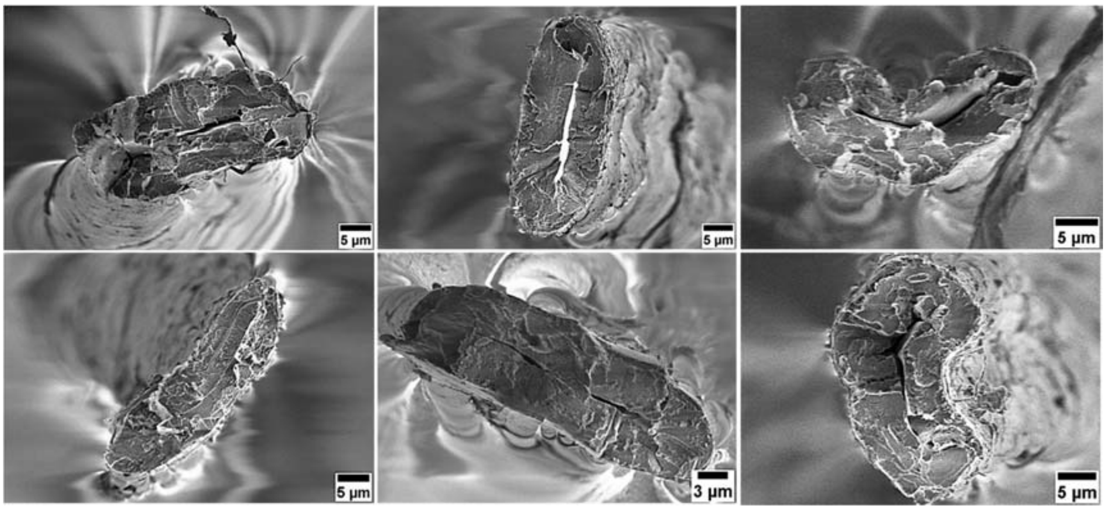

Figure S3. Examples of SEM images of the fractured surface of single ramie fibres show the flat and clear fracture end after tensile testing.

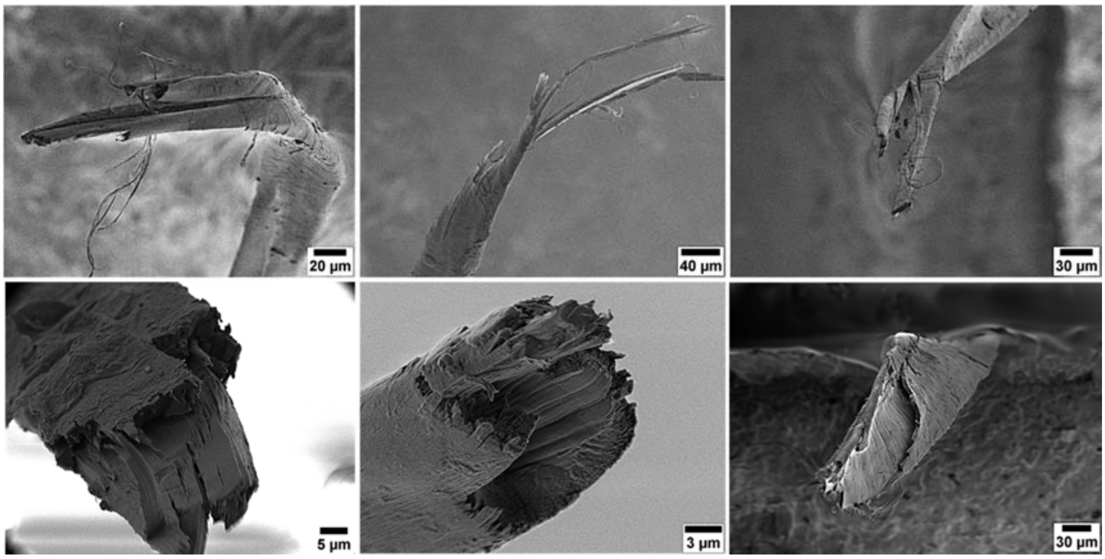

Figure S4. Examples of SEM images of the fractured surface of single ramie fibres show the irregular fracture end after tensile testing.

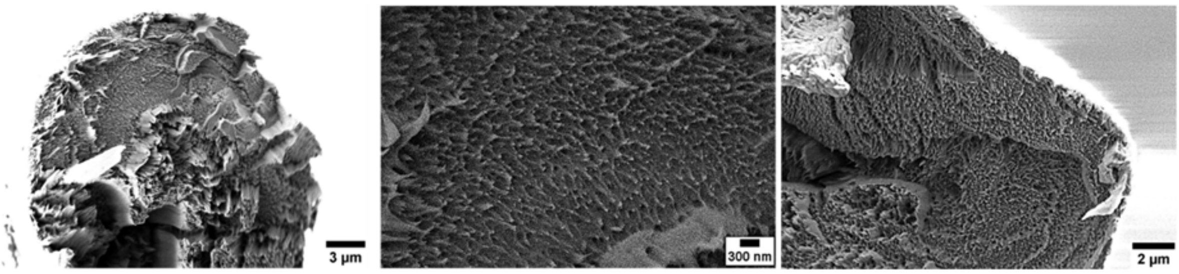

Figure S5. SEM images of the cell wall of ramie fibres after tensile testing show the microfibrils**.**

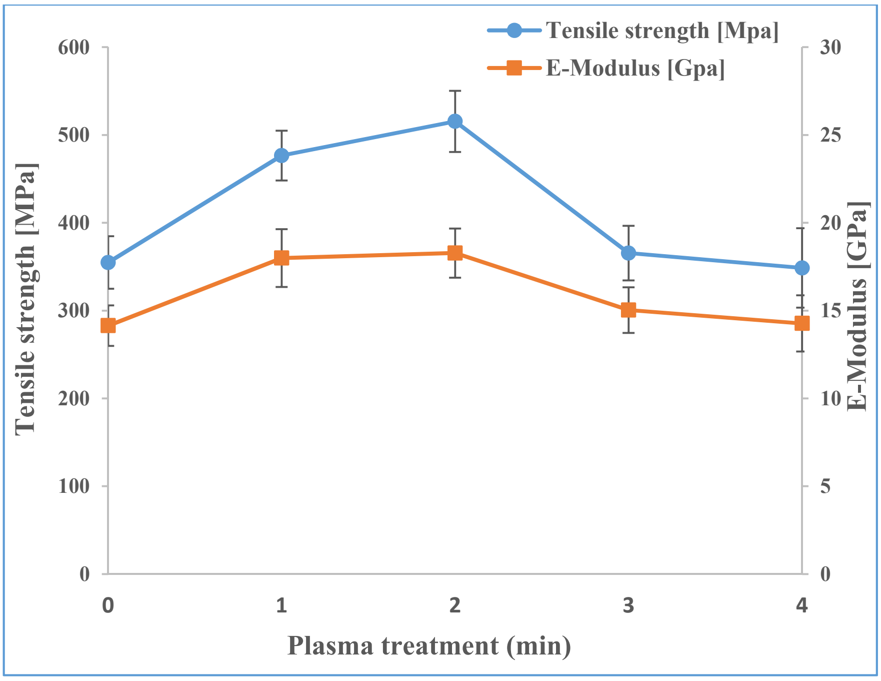

Figure S6. The tensile strength and Young's modulus of untreated and plasma treated fibres, values based on fibre diameter measurements (assuming circular cross section).

Table S1. The sample size for each fibre (untreated and plasma treated) that used to determine the tensile strength and Young's modulus using the actual cross sectional area.

Fibre	Sample size
**0 min**	5
**1 min**	10
**2 min**	9
**3 mon**	10
**4 min**	11
